# Supplementary material for: SALM4 negatively regulates NMDA receptor function and fear memory consolidation
Source: Commun Biol. 2021 Sep 29;4:1138. doi: 10.1038/s42003-021-02656-3 (PMC8481232; doi:10.1038/s42003-021-02656-3)
Supplement: Supplementary file 2 — Supplementary information [file 42003_2021_2656_MOESM2_ESM.pdf]

SALM4 negatively regulates NMDA receptor function and fear memory consolidation

Eunkyung Lie[1], Yeji Yeo[2], Eun-Jae Lee[3], Wangyong Shin[1], Kyungdeok Kim[1], Kyung Ah Han[4], Esther Yang[5], Tae-Yong Choi[6], Mihyun Bae[1], Suho Lee[1], Seung Min Um[2], Se-Young Choi[6], Hyun Kim[5], Jaewon Ko[4], and Eunjoon Kim[1,3]

[1]Center for Synaptic Brain Dysfunctions, Institute for Basic Science (IBS), Daejeon 34141, Korea; [2]Department of Biological Sciences, Korea Advanced Institute for Science and Technology (KAIST), Daejeon 34141, Korea; [3]Graduate School of Medical Science and Engineering, KAIST, Daejeon 34141, Korea; [4]Department of Brain and Cognitive Sciences, Daegu Gyeongbuk Institute of Science and Technology (DGIST), Hyeonpoong-Eup, Dalseong-Gun, Daegu 42988, Korea; [5]Department of Anatomy and Division of Brain Korea 21, Biomedical Science, College of Medicine, Korea University, Seoul 02841, Korea; [6]Department of Physiology and Neuroscience, Dental Research Institute, Seoul National University School of Dentistry, Seoul 03080, Korea

Supplementary figures and figure legends

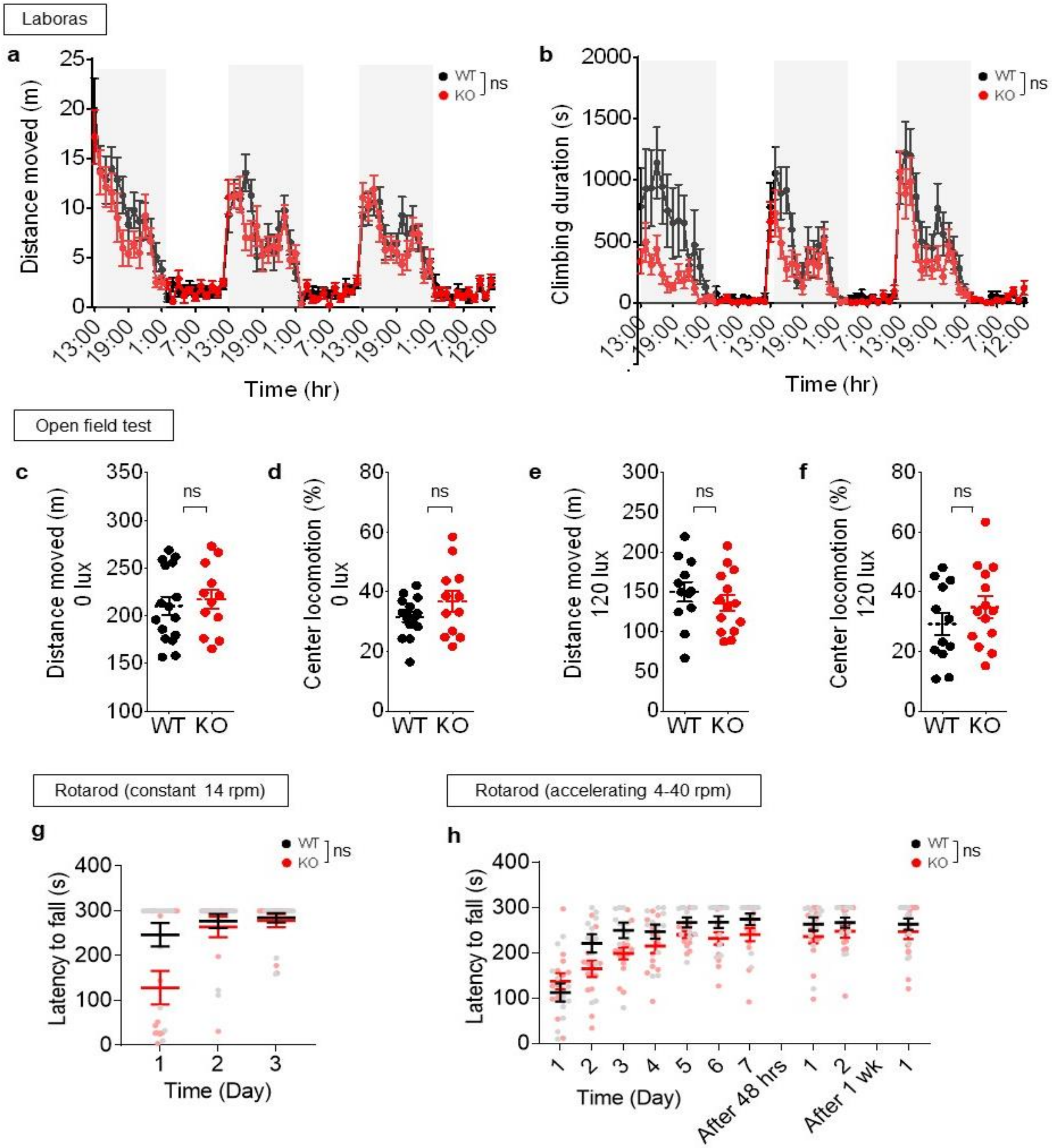

Supplementary Figure 1. *Lrfn3*$^{-/-}$ mice show normal locomotor activity in familiar and novel environments and normal motor coordination and learning.
(a and b) Normal locomotor and climbing activity of *Lrfn3*$^{-/-}$ mice (8–20 weeks) in Laboras cages, where mouse movements were monitored for three consecutive days. (n = 12 mice (WT) and 14 (KO); two-way RM-ANOVA (no genotype differences)).

(c–f) Normal locomotor activity of *Lrfn3*$^{-/-}$ mice (8–20 weeks) in the open-field test under complete-darkness (0 lux) and bright-light (120 lux) conditions. (n = 12 (WT) and 14 (KO) for 0 lux and 16 (WT) and 12 (KO) for 120 lux; ns, not significant, Student's t-test).

(g and h) Normal motor coordination and motor learning and memory in *Lrfn3*$^{-/-}$ mice (12–20 weeks) in the rotarod test. (n = 16 (WT) and 12 (KO) for constant speed (14 rpm); n = 12 (WT) and 14 (KO) for accelerating speed (4–40 rpm); ns, not significant (genotype difference), two-way RM-ANOVA).

Error bars represent the standard error of the mean.

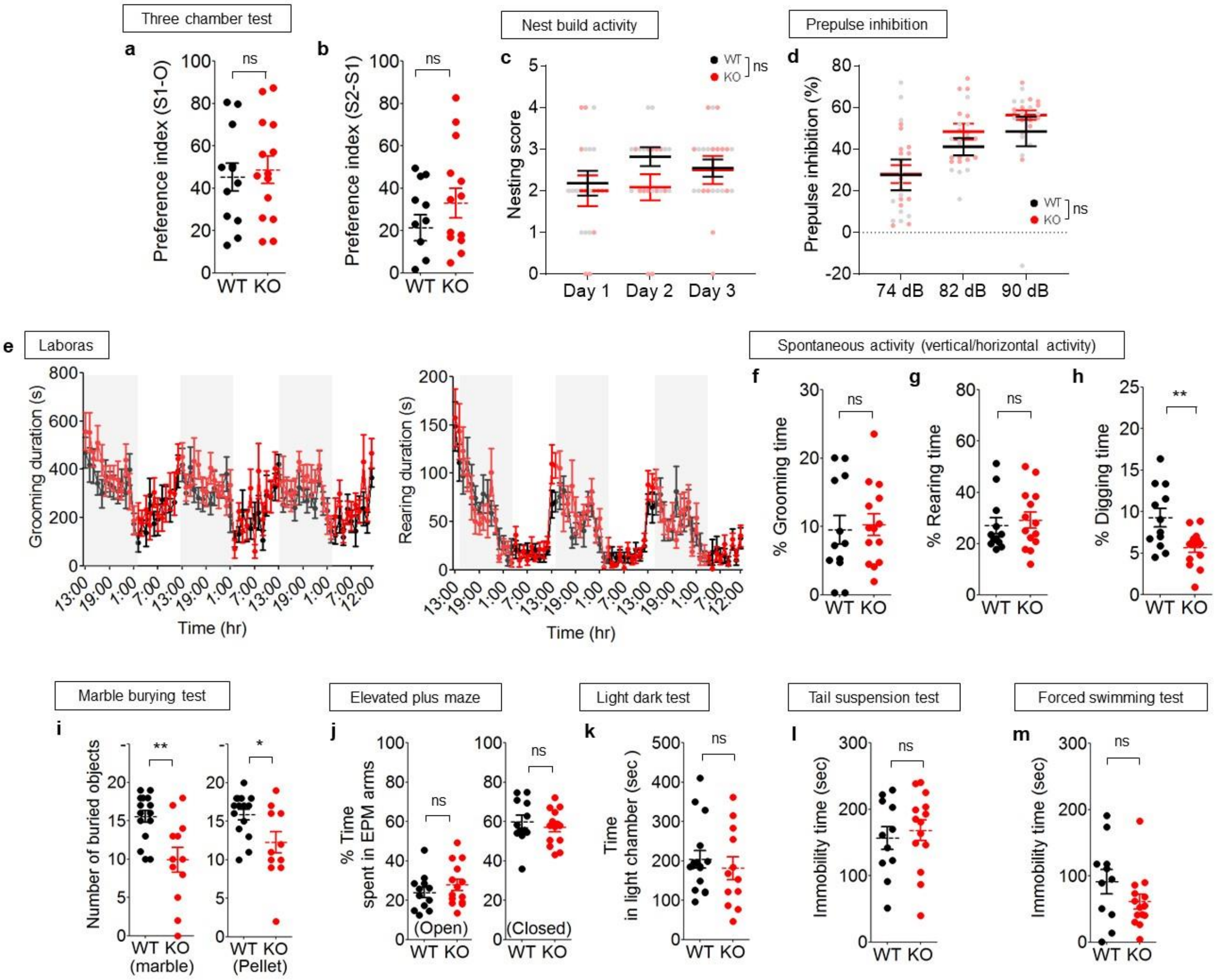

Supplementary Figure 2. *Lrfn3*$^{-/-}$ mice show normal social interaction, sensory-motor gating, spontaneous activity and anxiety/depression-related behavior, but moderately altered repetitive behavior.

(a and b) Normal social approach (a) and social-novelty recognition (b) in *Lrfn3*$^{-/-}$ mice (12–16 weeks) in the three-chamber test, as indicated by preference index (% time spent sniffing social target [S1] over total targets [S1 and O/Object], or novel social target [S2] over total targets [S1/familiar + S2/novel]). (n = 12 mice (WT) and 14 mice (KO); ns, not significant, Student's t-test).

(c) Normal nest building by *Lrfn3*$^{-/-}$ mice (12–20 weeks). (n =11 (WT) and 14 (KO); ns, not significant, two-way RM-ANOVA).

(d) Normal sensory-motor gating in *Lrfn3*$^{-/-}$ mice (10–12 weeks) in the prepulse-inhibition test. (n =11 (WT) and 14 (KO); ns, not significant, Student's t-test).

(e–i) Normal self-grooming and rearing but reduced digging (e–g) and marble/food pellet burying (h) by *Lrfn3*$^{-/-}$ mice in home cages (8–16 weeks for self-grooming/rearing/digging; 12–16 weeks for marble/food pellet burying). (n = 12 (WT)

and 14 (KO) for self-grooming/digging and n = 16 (WT) and 12 (KO) for marble burying, two-way RM-ANOVA (e); *$p < 0.05$, **$p < 0.01$, ns, not significant, Student's t-test (f–i)).

(j) Normal anxiety-like behavior of *Lrfn3*$^{-/-}$ mice (10–14 weeks) in the elevated plus-maze test, as indicated by time spent in open/closed arms. (n = 12 (WT) and 14 (KO); ns, not significant, Student's t-test).

(k) Normal anxiety-like behavior of *Lrfn3*$^{-/-}$ mice (10–16 weeks) in the light-dark test, as indicated by time spent in the light chamber. (n = 16 (WT) and 12 (KO); ns, not significant, Student's t-test).

(l) Normal depression-like behavior of *Lrfn3*$^{-/-}$ mice (12–20 weeks) in the tail suspension test. (n =11 (WT) and 14 (KO); ns, not significant, Student's t-test).

(m) Normal depression-like behavior of *Lrfn3*$^{-/-}$ mice (12–20 weeks) in the forced swim test. (n =11 (WT) and 14 (KO); ns, not significant, Student's t-test).

Error bars represent the standard error of the mean.

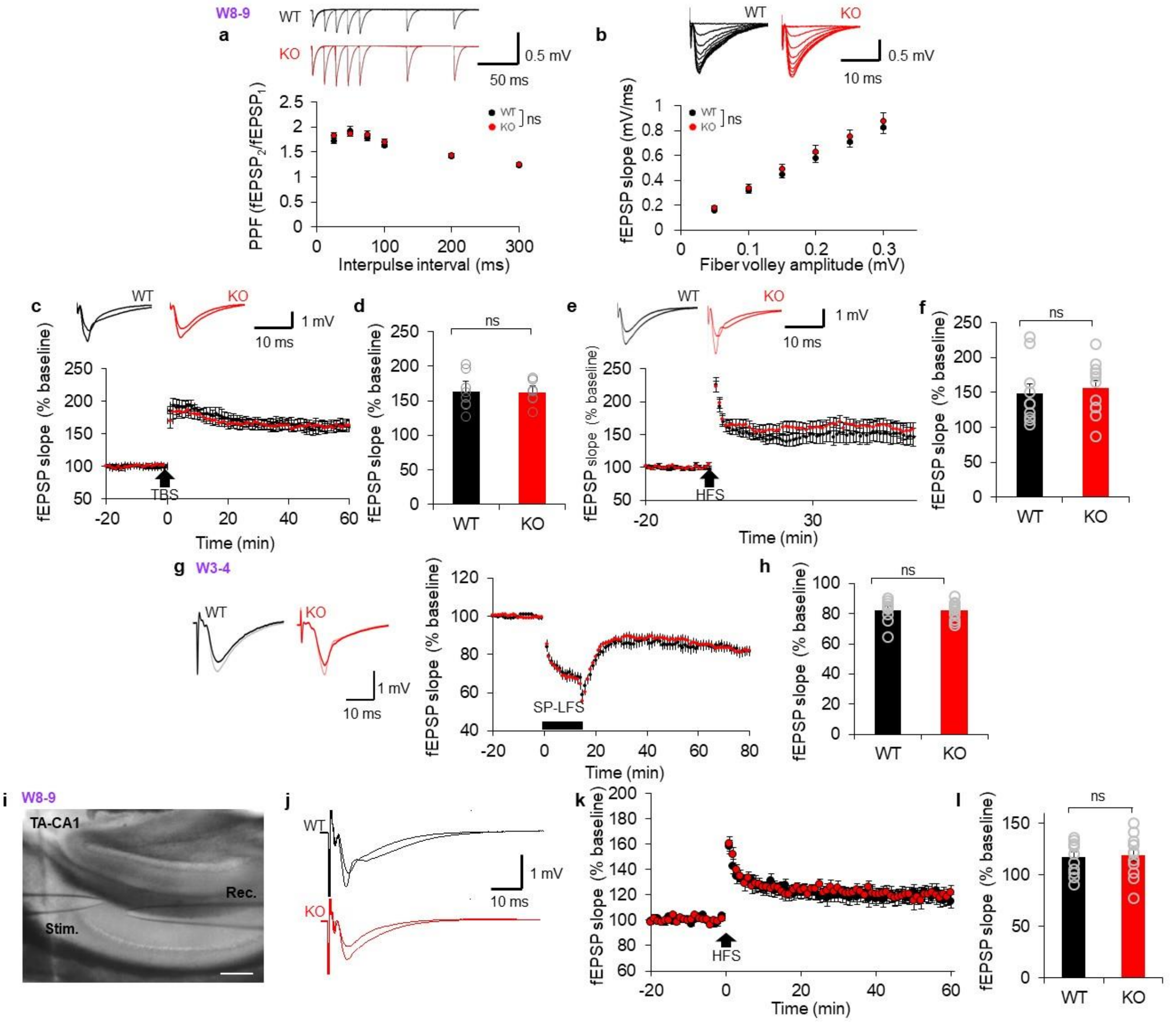

Supplementary Fig. 3. *Lrfn3*$^{-/-}$ mice show normal paired-pulse facilitation, basal excitatory transmission, TBS-LTP, HFS-LTP, and LFS-LTD in the hippocampus.
(a) Normal paired-pulse facilitation at SC-CA1 synapses in the hippocampus of *Lrfn3*$^{-/-}$ mice (8–9 weeks). (n = 9 slices from 3 mice (WT), and 9, 3 (KO); ns, not significant, Student's t-test).
(b) Normal excitatory synaptic transmission at SC-CA1 synapses in the *Lrfn3*$^{-/-}$ (KO) hippocampus (8–9 weeks), as indicated by input-output curve plotting fEPSP slopes against fiber volley amplitudes. (n = 16, 5 (WT), and 19, 5 (KO); ns, not significant, Student's t-test).
(c–h) Normal TBS-LTP (c and d), HFS-LTP (e and f), and LFS-LTD (g and h) at SC-CA1 synapses in the *Lrfn3*$^{-/-}$ (KO) hippocampus (8–9 weeks for TBS-LTP and HFS-LTP and 3–4 weeks for LFS-LTD). (n = 8, 3 (WT), 7, 3 (KO) for TBS-LTP, n = 10, 6 (WT), 11, 7 (KO) for HFS-LTP, n = 10, 5 (WT), 12, 4 (KO) for LFS-LTD, Student's t-test (last 10 min)).

(i–l) Normal HFS-LTP at TA-CA1 synapses in the distal region of CA1 dendrites (8–9 weeks). The locations of stimulating and recording electrodes are indicated in a sample slice image. (n = 11, 3 (WT) and 12, 3 (KO), ns, not significant, Student's t-test (last 10 min)). Scale bar, 200 µm.
Error bars represent the standard error of the mean.

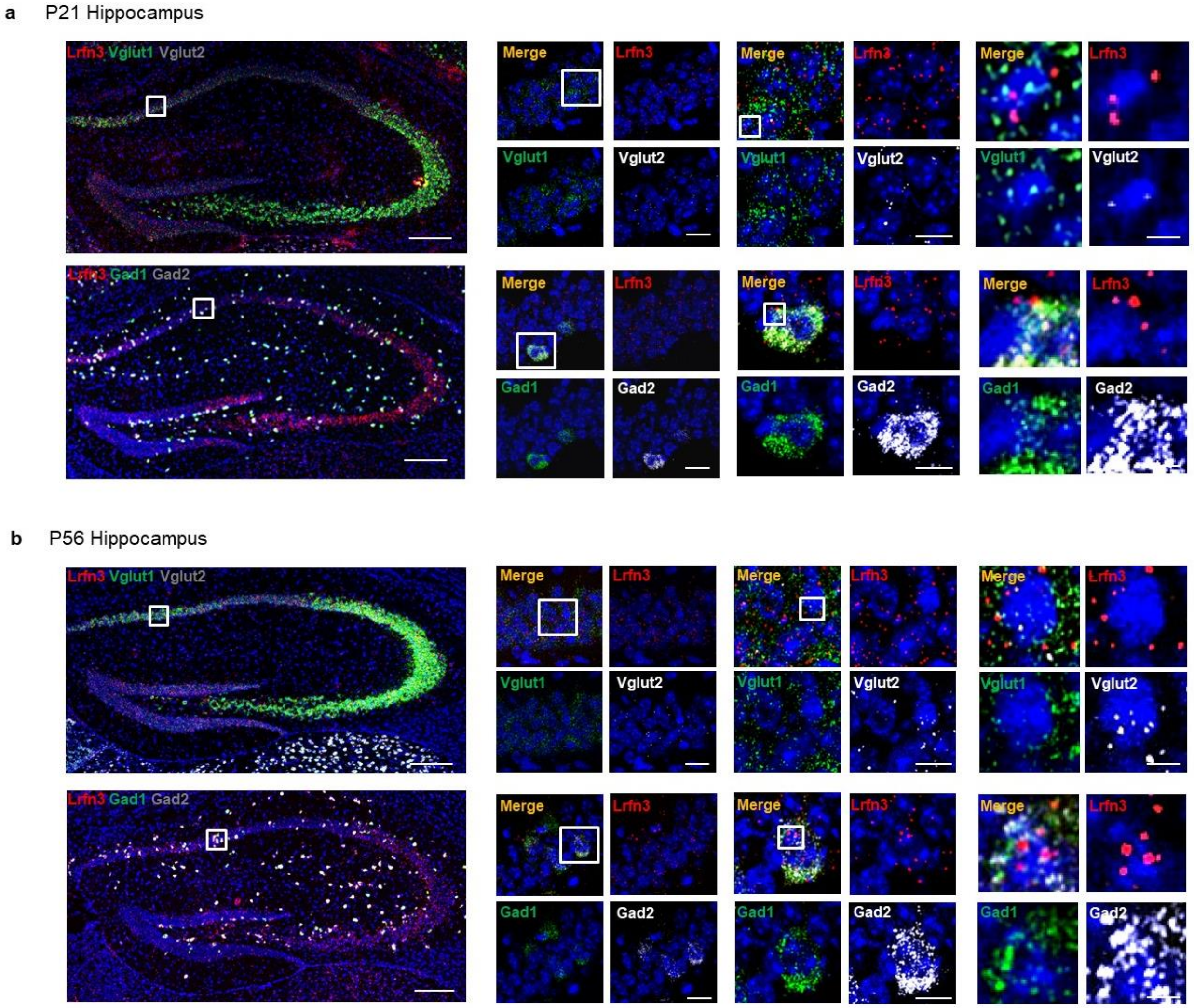

Supplementary Fig. 4. Lrfn3/SALM4 mRNAs detected in glutamate and GABAergic neurons in hippocampal mouse brain regions.

(a) Expression of Lrfn3/SALM4 mRNA in Vglut1/2-positive glutamatergic neurons (upper panels) and Gad1/2-positive GABAergic neurons (lower panels) in the hippocampal formation of P21 (a) and P56 (b) mouse brains. Coronal brain sections were probed by double fluorescence in situ hybridization (FISH) for Lrfn3/SALM4 and Vglut1/2 or Gad1/2, and counterstained with DAPI (nuclear stain; blue). Images on the right side represent enlarged images of the single cells in the insets in the CA1 region on the left. Note that Lrfn3/SALM4 mRNAs are detected in various regions of the hippocampal formation (CA1, CA3, and dentate gyrus) and colocalized with both glutamatergic and GABAergic neurons in these regions. Scale bar, 200, 20, 10, and 2 μm.

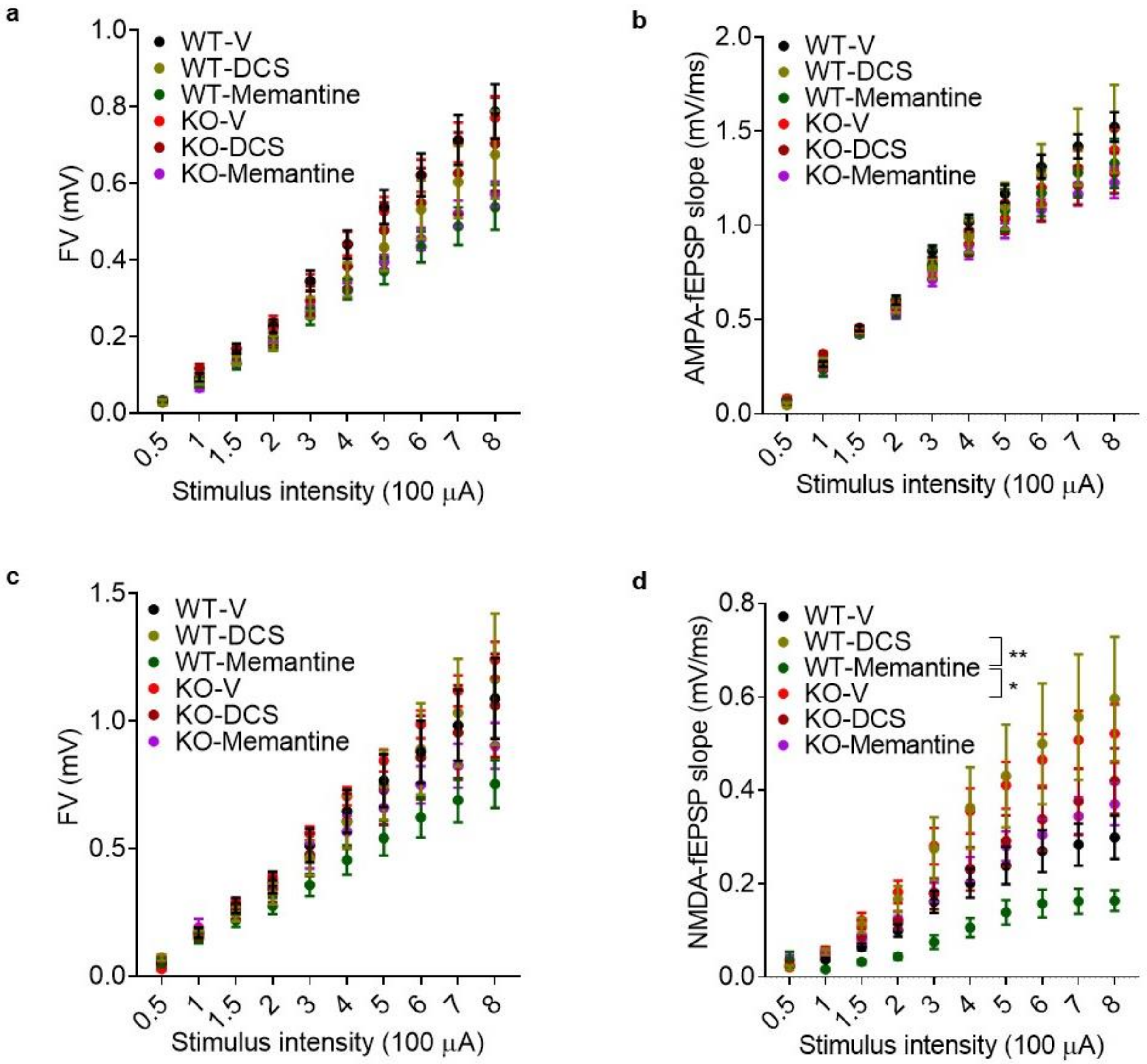

Supplementary Fig. 5. Differential effects of NMDAR modulators on NMDAR and AMPAR functions at *Lrfn3*$^{-/-}$ hippocampal synapses.
(a–d) Changes in NMDAR and AMPAR components at SC-CA1 synapses induced by chronic treatment of adult *Lrfn3*$^{-/-}$ mice (P56–91) with DCS (20 mg/kg/d; 7 days; i.p.), memantine (10 mg/kg/d; 7 days; i.p.) or vehicle (V), as shown by initial slopes of fiber volley values or fEPSPs (NMDAR or AMPAR) plotted against stimulus intensities. Simplified versions of the six-group comparisons in panels (a) and (c) are shown in panels (b) and (d). (n = 8 slices from 6 mice (WT), and 9, 5 (KO); *$p < 0.05$, **$p < 0.01$, two-way RM-ANOVA with Tukey's test).
Error bars represent the standard error of the mean.

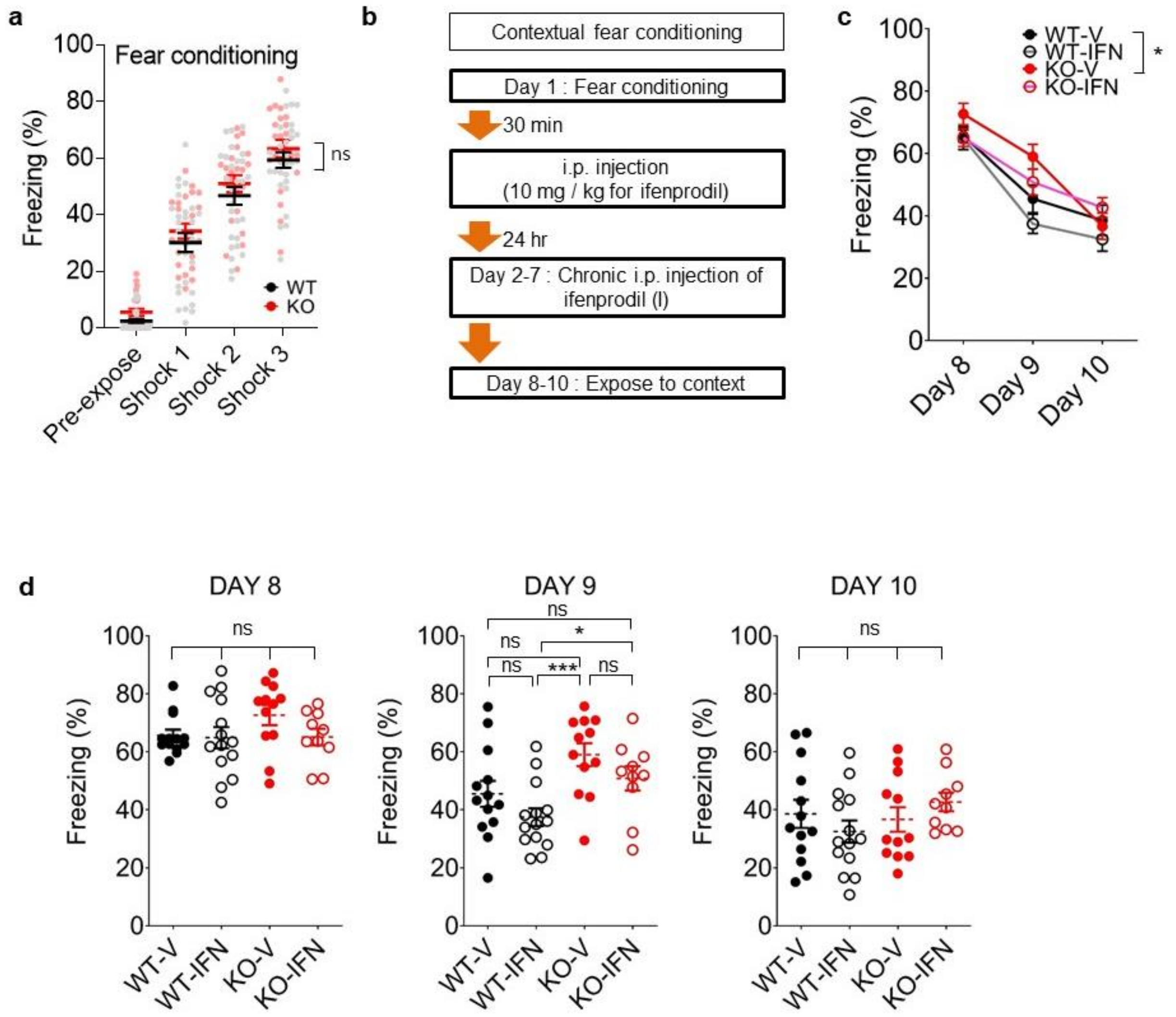

Supplementary Fig. 6. Ifenprodil fails to rescue enhanced fear memory consolidation in *Lrfn3*$^{-/-}$ mice.

(a–g) Changes in the levels of contextual fear memory consolidation in adult *Lrfn3*$^{-/-}$ mice (2–6 months) after normal fear memory acquisition (a), chronic treatment with ifenprodil (10 mg/kg/d; 7 days; i.p.), or vehicle (V), as shown by freezing levels during days 8–10. After initial contextual fear memory acquisition (a), WT and KO mice were divided into four groups (WT-V, -WT-IFN/ifenprodil, KO-V, and KO-IFN) for chronic drug treatment. (n = 13 mice (WT-V), 14 (WT-IFN), 12 (KO-V), and 10 (KO-IFN), $*p < 0.05$, $***p < 0.001$, ns, not significant, two-way RM-ANOVA with Bonferroni test). Error bars represent the standard error of the mean.

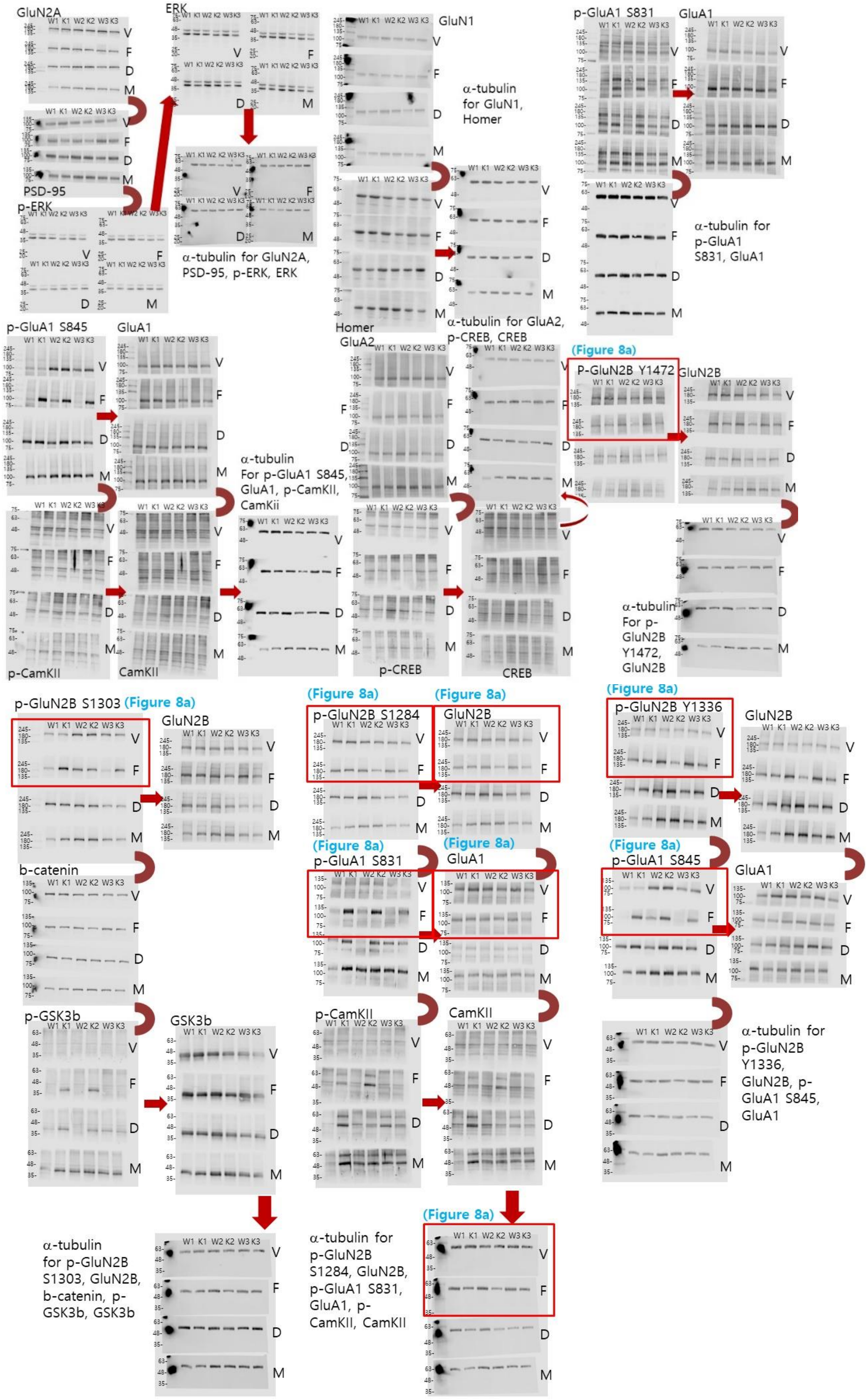
GluN2A
ERK
GluN1
p-GluA1 S831
GluA1
PSD-95
p-ERK
α-tubulin for GluN1, Homer
α-tubulin for GluN2A, PSD-95, p-ERK, ERK
α-tubulin for p-GluA1 S831, GluA1
Homer
GluA2
α-tubulin for GluA2, p-CREB, CREB
p-GluA1 S845
(Figure 8a)
P-GluN2B Y1472
GluN2B
α-tubulin For p-GluA1 S845, GluA1, p-CamKII, CamKii
p-CamKII
CamKII
p-CREB
CREB
α-tubulin For p-GluN2B Y1472, GluN2B
p-GluN2B S1303
p-GluN2B S1284
p-GluN2B Y1336
b-catenin
p-GluA1 S831
p-GluA1 S845
p-GSK3b
GSK3b
α-tubulin for p-GluN2B Y1336, GluN2B, p-GluA1 S845, GluA1
α-tubulin for p-GluN2B S1303, GluN2B, b-catenin, p-GSK3b, GSK3b
α-tubulin for p-GluN2B S1284, GluN2B, p-GluA1 S831, GluA1, p-CamKII, CamKII
W1 K1 W2 K2 W3 K3
V
F
D
M

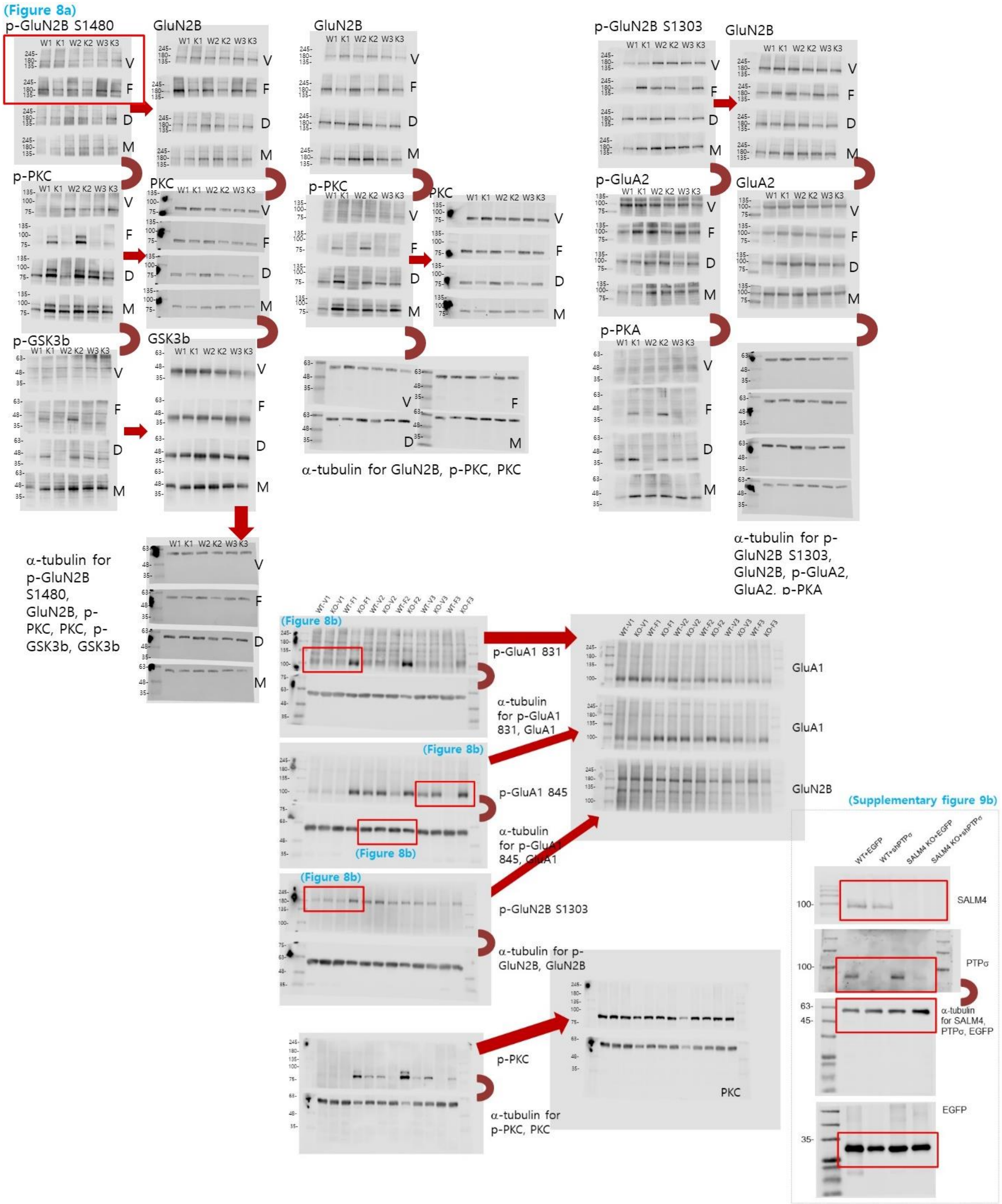

Supplementary Fig. 7. Full immunoblot images of fluoxetine-dependent changes in GluN2B and GluA1 phosphorylation in WT and *Lrfn3*$^{-/-}$ mice.
The reason why there is only one loading control (alpha-tubulin) for a couple of proteins is that a full-length PAGE gel was horizontally cut into several pieces to immunoblot for several target proteins where one control blot was generated. The semicircular rings in

the raw full-length immunoblot images indicate the junctions between the horizontally separated full-length PAGE gels.

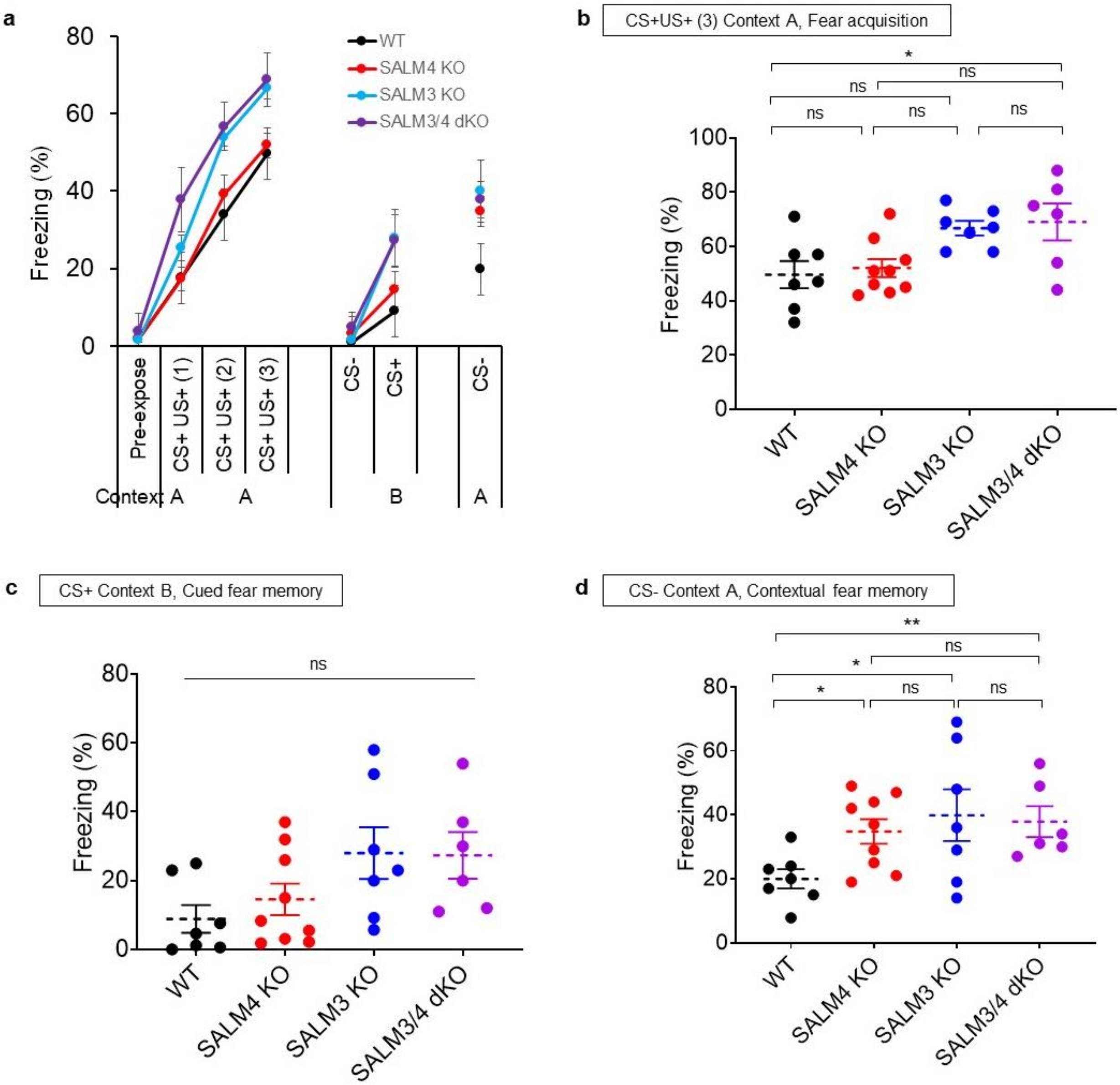

Supplementary Fig. 8. Enhanced fear memory consolidation in *Lrfn3*$^{-/-}$ mice lacking SALM4 does not likely involve SALM3.

(a–d) Comparison of fear memory acquisition, 24-hr cued fear memory retention, and 48-hr contextual fear memory retention in mice lacking SALM4 (*Lrfn3*$^{-/-}$; SALM4 KO), SALM3 (*Lrfn4*$^{-/-}$; SALM3 KO), and both SALM4 and SALM 3 (*Lrfn3*$^{-/-}$;*Lrfn4*$^{-/-}$; SALM3/4 dKO). Single and dKO mice conditioned to both context A and a tone cue were exposed to the tone cue in context B (cued fear memory) on day 2 and in context A on day 3 (contextual fear memory). (n = 7 mice (WT), 9 (SALM4 KO), 7 (SALM3 KO), 6 (SALM3/4 dKO); *$p < 0.05$, **$p < 0.01$, Student's t-test).
Error bars represent the standard error of the mean.

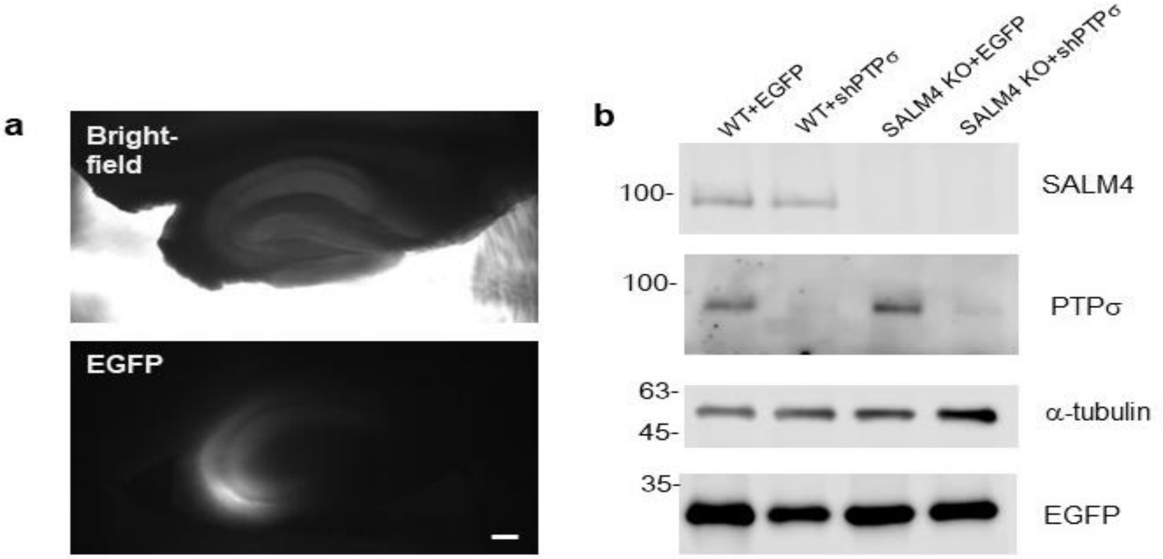

Supplementary Fig. 9. AAV-mediated knockdown of PTPσ in WT and *Lrfn3*$^{-/-}$ hippocampal CA3 region.

(a and b) AAV-U6-GFP (control) or AAV-U6-GFP-shPTPσ virus particles were injected into the hippocampal CA3 region of WT and *Lrfn3*$^{-/-}$ mice (2–6 months). After 2 weeks, these mice were tested for shRNA expression through GFP signals in the CA3 region (a) [and the knockdown of target PTPσ proteins in the CA3 region by immunoblot analysis of CA3 lysates (b). Scale bar, 200 μm.

a

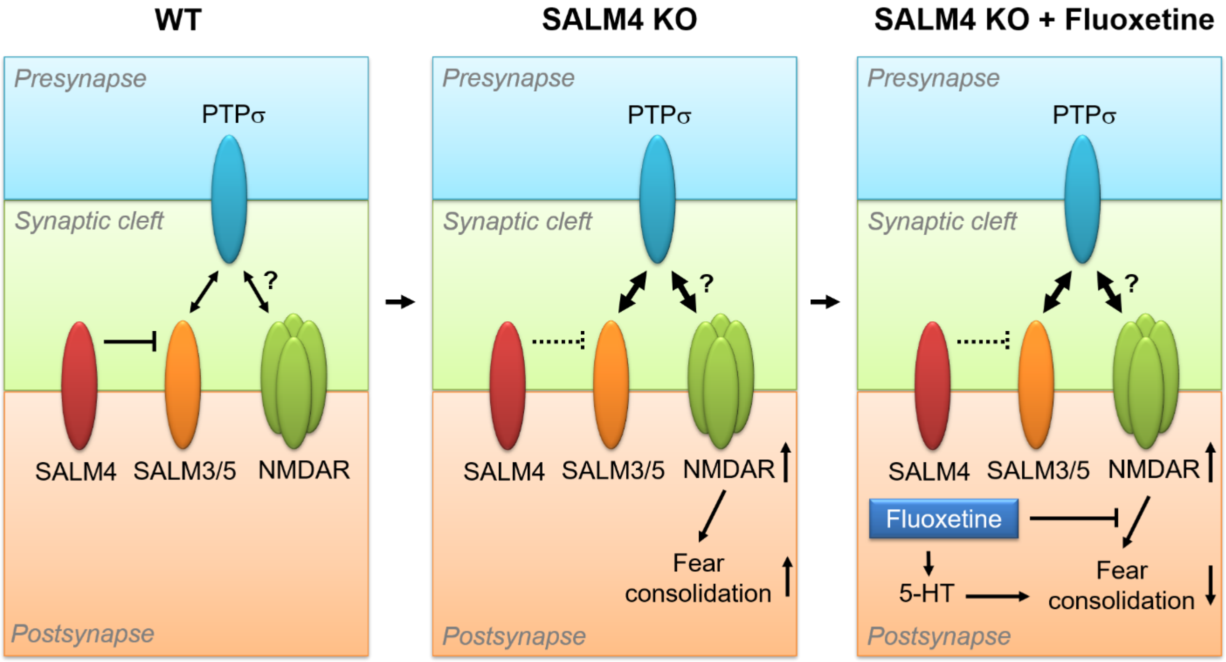

Supplementary Fig. 10. Working model for NMDAR hyperfunction and fluoxetine-dependent rescue in *Lrfn3*$^{-/-}$ mice.

(a) In WT mice, postsynaptic SALM4 cis-interacts with and inhibits postsynaptic SALM3/5 to suppress their trans-synaptic interactions with presynaptic PTPσ, known to promote excitatory synapse development. PTPσ also promotes postsynaptic NMDAR responses through trans-synaptic mechanisms that are not clear yet. When SALM4 is deleted, SALM3/5 may be disinhibited and more strongly interact with PTPσ to promote excitatory synapse development, leading to abnormal NMDAR hyperactivity and enhanced fear memory consolidation. SALM3 (but not SALM5) is less likely to contribute to to this hypothesis, considering the lack of fear memory consolidation by the SALM3/4 dKO. Fluoxetine may inhibit NMDAR activity as a direct antagonist or indirectly modulate the serotonin/5-HT system to normalize the NMDAR hyperactivity and enhanced fear memory consolidation in SALM4-mutant (*Lrfn3*$^{-/-}$) mice.
