# Supplementary material for: SALM4 negatively regulates NMDA receptor function and fear memory consolidation
Source: Commun Biol. 2021 Sep 29;4:1138. doi: 10.1038/s42003-021-02656-3 (PMC8481232; doi:10.1038/s42003-021-02656-3)
Supplement: Supplementary file 3 — Description of Supplementary Files [file 42003_2021_2656_MOESM3_ESM.pdf]

Description of Supplementary Files

File name: Supplementary data 1
Description: Source data for graphs and charts.

File name: Supplementary data 2
Description: Statistical details including mouse age and n numbers, statistical methods, and statistical results are described.
