# Supplementary material for: SALM4 negatively regulates NMDA receptor function and fear memory consolidation
Source: Commun Biol. 2021 Sep 29;4:1138. doi: 10.1038/s42003-021-02656-3 (PMC8481232; doi:10.1038/s42003-021-02656-3)
Supplement: Supplementary file 6 — Reporting Summary [file 42003_2021_2656_MOESM6_ESM.pdf]

nature research

Corresponding author(s): Eunjoon Kim

Last updated by author(s): Aug 21, 2021

# Reporting Summary

Nature Research wishes to improve the reproducibility of the work that we publish. This form provides structure for consistency and transparency in reporting. For further information on Nature Research policies, see Authors & Referees and the Editorial Policy Checklist .

## Statistics

For all statistical analyses, confirm that the following items are present in the figure legend, table legend, main text, or Methods section.

| n/a | Confirmed | |
|---|---|---|
| ☐ | ☒ | The exact sample size ($n$) for each experimental group/condition, given as a discrete number and unit of measurement |
| ☐ | ☒ | A statement on whether measurements were taken from distinct samples or whether the same sample was measured repeatedly |
| ☐ | ☒ | The statistical test(s) used AND whether they are one- or two-sided<br>*Only common tests should be described solely by name; describe more complex techniques in the Methods section.* |
| ☒ | ☐ | A description of all covariates tested |
| ☒ | ☐ | A description of any assumptions or corrections, such as tests of normality and adjustment for multiple comparisons |
| ☐ | ☒ | A full description of the statistical parameters including central tendency (e.g. means) or other basic estimates (e.g. regression coefficient) AND variation (e.g. standard deviation) or associated estimates of uncertainty (e.g. confidence intervals) |
| ☐ | ☒ | For null hypothesis testing, the test statistic (e.g. $F$, $t$, $r$) with confidence intervals, effect sizes, degrees of freedom and $P$ value noted<br>*Give P values as exact values whenever suitable.* |
| ☒ | ☐ | For Bayesian analysis, information on the choice of priors and Markov chain Monte Carlo settings |
| ☒ | ☐ | For hierarchical and complex designs, identification of the appropriate level for tests and full reporting of outcomes |
| ☒ | ☐ | Estimates of effect sizes (e.g. Cohen's $d$, Pearson's $r$), indicating how they were calculated |

*Our web collection on statistics for biologists contains articles on many of the points above.*

## Software and code

Policy information about availability of computer code

| | |
|---|---|
| Data collection | LI-COR Odyssey (version) Fc<br>pClamp 10.1 (Molecular Devices)<br>Multiclamp Commander 700B (Molecular Devices)<br>Digidata 1550 (Molecular Devices)<br>LABORAS (METRIS)<br>FreezeFrame3 (Coulbourn Instruments)<br>LSM780 (Carl Zeiss)<br>TCS SP8 Dichroic/CS (Leica) |
| Data analysis | LABORAS (METRIS)<br>FreezeFrame3 (Coulbourn Instruments)<br>Ethovision XT 10.1 (Noldus)<br>Clampfit 10.7 (Molecular Devices)<br>Image Studio Lite Ver. 5.2.5<br>GraphPad Prism 8.0<br>Image J |

For manuscripts utilizing custom algorithms or software that are central to the research but not yet described in published literature, software must be made available to editors/reviewers. We strongly encourage code deposition in a community repository (e.g. GitHub). See the Nature Research guidelines for submitting code & software for further information.

## Data

Policy information about availability of data

All manuscripts must include a data availability statement. This statement should provide the following information, where applicable:

- Accession codes, unique identifiers, or web links for publicly available datasets
- A list of figures that have associated raw data
- A description of any restrictions on data availability

The data supporting the findings of this study are available within the Supplementary Table 1. Other source data related to the study are available from the corresponding author upon reasonable request.

# Field-specific reporting

Please select the one below that is the best fit for your research. If you are not sure, read the appropriate sections before making your selection.

☒ Life sciences ☐ Behavioural & social sciences ☐ Ecological, evolutionary & environmental sciences

For a reference copy of the document with all sections, see nature.com/documents/nr-reporting-summary-flat.pdf

# Life sciences study design

All studies must disclose on these points even when the disclosure is negative.

| | |
|---|---|
| Sample size | No statistical methods were used to predetermine sample size. Estimates were made based on our previous experience, experimental approach, availability and feasibility required to obtain statistically significant results. For detail sample size please see figure legends. |
| Data exclusions | Outliers were excluded based on the results of the ROUT test (Q = 1%). |
| Replication | All experiments were replicated through multiple cohort/mice analysis, where applicable. All replication attempts were successful. In the case of single experiments (multiple samples in a single experiment), such experimental designs were described in the legends |
| Randomization | Mice were allocated into specific cohorts at random, except genotype per cage post-weaning was set at a 1:1 ratio for WT vs KO. Male cohorts were caged separately from female cohorts if weaned. Pup and juvenile mice were not weaned and not separated, with experiments being performed with whole cages 'as is'. |
| Blinding | All experimenters were blind to the genotype of the mice (sex could not be occluded from the experimenter, due to obviousness of the features). All analyses were performed in a blind manner. Cohorts were grouped at random at time of weaning. |

# Reporting for specific materials, systems and methods

We require information from authors about some types of materials, experimental systems and methods used in many studies. Here, indicate whether each material, system or method listed is relevant to your study. If you are not sure if a list item applies to your research, read the appropriate section before selecting a response.

## Materials & experimental systems

| n/a | Involved in the study | |
|---|---|---|
| ☐ | ☒ | Antibodies |
| ☒ | ☐ | Eukaryotic cell lines |
| ☒ | ☐ | Palaeontology |
| ☐ | ☒ | Animals and other organisms |
| ☒ | ☐ | Human research participants |
| ☒ | ☐ | Clinical data |

## Methods

| n/a | Involved in the study | |
|---|---|---|
| ☒ | ☐ | ChIP-seq |
| ☒ | ☐ | Flow cytometry |
| ☒ | ☐ | MRI-based neuroimaging |

## Antibodies

| | |
|---|---|
| Antibodies used | Gp polyclonal anti-PTPsigma (made in-house #2135, 1:1000)<br>Rb polyclonal anti-Homer (made in-house # 1133, 1:1000)<br>Rb polyclonal anti-GluA1 (made in-house # 1193, 1:1000)<br>Rb polyclonal anti-phospho-GluA1 Ser831 (Millipore, Cat # AB5847, lot # aliquot used, 1:500)<br>Rb polyclonal anti-phospho-GluA1 Ser845 (Millipore, Cat # AB5849, lot # aliquot used, 1:500)<br>Rb polyclonal anti-GluA2 (made in-house # 1195, 1:1000)<br>Rb polyclonal anti-phospho-GluA2 (Tyr869/Tyr873/Tyr876) (CST, Cat # 3921, lot # 2, 1:500)<br>Gp polyclonal anti-CamKII (made in-house # 1300, 1:1000) |

Rb polyclonal anti-phospho CamKII (T286) (abcam, Cat # ab32678, lot # GR3247497-1, 1:1000)
Rb polyclonal anti-PKA alpha+beta (catalytic subunits-N term) (abcam, Cat # ab71764, lot # aliquot used, 1:1000)
Mo monoclonal anti-PKC alpha (BD biosciences, Cat # 610108, lot # aliquot used, 1:1000)
Rb polyclonal anti-phospho-PKC (pan) (betaII Ser660) (CST, Cat # 9371, lot # aliquot used, 1:500)
Rb polyclonal anti-p44/42 MAPK (Erk1/2) (CST, Cat # 9102, lot # 23, 1:500)
Rb polyclonal anti-phospho-p44/42 MAPK (Erk1/2) (Thr202/Tyr204) (CST, Cat # 9101, lot # 31, 1:500)
Rb monoclonal anti-CREB (48H2) (CST, Cat # 9197, lot # 16, 1:500)
Rb monoclonal anti-phospho-CREB (Ser133) (87G3) (CST, Cat # 9198, lot # 10, 1:500)
Rb monoclonal anti-GSK3beta (27C10) (CST, Cat # 9315, lot # 14, 1:500)
Rb polyclonal anti-phospho-GSK3beta (Ser9) (CST, Cat # 9336, lot # 13, 1:500)
Rb polyclonal anti-phospho-GluN2B (Tyr1472) (CST, Cat # 4208, lot # 3, 1:500)
Rb polyclonal anti-phospho-GluN2B (Ser1284) (CST, Cat # 5355, lot # aliquot used, 1:500)
Rb polyclonal anti-phospho-GluN2B (Tyr1472) (CST, Cat # 4208, lot # 3, 1:500)
Rb polyclonal anti-phospho-GluN2B (Tyr1336) (abcam, Cat # ab138664, lot # aliquot used, 1:500)
Rb polyclonal anti-phospho-GluN2B (Ser1480) (abcam, Cat # ab73014, lot # aliquot used, 1:500)
Rb polyclonal anti-phospho-GluN2B (Ser1303) (Milipore, Cat # 07-398, lot # aliquot used, 1:500)
Rb polyclonal anti-GluN2B (extracellular) (alomone labs, Cat # AGC-003, lot # aliquot used, 1:1000)
Rb polyclonal anti-GluN2A (Milipore, Cat # 07-632, lot # aliquot used, 1:1000)
Mo monoclonal anti-GluN1/NR1 (Neuromab, Cat # 75-272, lot # aliquot used, 1:500)
Mo monoclonal anti-beta-catenin (BD Transduction, Cat # 610154, lot # aliquot used, 1:1000)
Mo monoclonal anti-alpha-tubulin (DSHB, Cat # 12G10, lot # aliquot used, 1:10000)
Gp polyclonal anti-GFP (made in-house #1998, 1:1000)

Validation

All commercial antibodies and antibodies made in-house have been validated and published, with relevant information existing in the pertaining website and paper (Lie et al., Nature Communications, 2016).

## Animals and other organisms

Policy information about studies involving animals; ARRIVE guidelines recommended for reporting animal research

Laboratory animals

C56BL/6J strain were used as background of all wildtype/mutant mouse used in the study. Only male mice were used for production of data. Behavioral studies involved adult mice (> postnatal day 70), while all other data involved juvenile (postnatal day 20~28) mice.

Wild animals

Study did not involve wild animals

Field-collected samples

Study did not involve samples collected from the field

Ethics oversight

All animals used in this study were maintained and procedures performed in accord with the Requirements of Animal Research at KAIST. Experimental procedures were approved by the Committee on Animal Research at KAIST (KA2016-32, KA2020-96).

Note that full information on the approval of the study protocol must also be provided in the manuscript.
